# Identification of markers for neurescence through transcriptomic profiling of postmortem human brains

**DOI:** 10.21203/rs.3.rs-5903682/v1

**Published:** 2025-04-09

**Authors:** Shiva Kazempour Dehkordi, Sogand Sajedi, Amirreza Heshmat, Miranda E. Orr, Habil Zare

**Affiliations:** The University of Texas Health Science Center at San Antonio; The University of Texas MD Anderson Cancer Center; The University of Texas MD Anderson Cancer Center; Washington University School of Medicine; The University of Texas Health Science Center at San Antonio

## Abstract

Neuronal senescence (i.e., neurescent) is an important hallmark of aging and neurodegeneration, but it remains poorly characterized in the human brain due to the lack of reliable markers. This study aimed to identify neurescent markers based on single-nucleus transcriptome data from postmortem human prefrontal cortex. Using an eigengene approach, we integrated three gene panels: a) SenMayo, b) Canonical Senescence Pathway (CSP), and c) Senescence Initiating Pathway (SIP), to identify neurescent signatures. We found that paired markers outperform single markers; for instance, by combining *CDKN2D* and *ETS2* in a decision tree, a high accuracy of 99% and perfect specificity (100%) were achieved in distinguishing neurescent. Differential expression analyses identified 324 genes that are overexpressed in neurescent. These genes showed significant associations with important neurodegeneration-related pathways including Alzheimer’s disease, Parkinson’s disease, and Huntington’s disease. Interestingly, several of these overexpressed genes are linked to mitochondrial dysfunction and cytoskeletal dysregulation. These findings provide valuable insights into the complexities of neurescent, emphasizing the need for further exploration of histologically viable markers and validation in broader datasets.

## Introduction

Cellular senescence is a complex, multi-step process characterized by stable cell cycle arrest^1^, changes in cell morphology^2,3^, changes in gene expression^4–8^, and a pro-inflammatory secretory phenotype^9,10^. Senescence was first identified in fibroblasts^11^, is heterogeneous in tissues throughout the body^12–14^, and has been studied in the context of the human brain^15,16^. Increasing evidence implicates cellular senescence in brain aging and its links to neurodegenerative disorders ^17,18^, including Alzheimer’s disease (AD)^19,20^. The presence of senescent cells triggers a pro-inflammatory environment^21^ and contributes to neuron loss, tissue dysfunction and cognitive impairment in animal models of AD^22,23^. Recent studies using AD mouse models and human postmortem brain tissues directly demonstrate that neuronal senescence associated with tau pathology drives neuronal loss and neurodegeneration^18^, and that senescent human astrocytes can induce neuronal death through excitotoxicity in co-culture systems^24^. In the human brain, senescent cells have been associated with the known AD pathologies e.g., amyloid plaques and neurofibrillary tangles^18,25,26^.

Despite their significance, defining and identifying senescent cells in the human brain remains challenging due to their heterogeneous nature^27^. Although reliable markers have been utilized to identify senescent cells in specific tissues such as adipose tissue, retinal endothelial cells, and fibroblast^12,28–33^, a universally applicable set of senescence markers across diverse tissues remain poorly defined. This challenge is especially pronounced in neurons, where historically defined senescence markers have not been robustly validated ^34^. This knowledge gap has limited our understanding of when senescent cells first appear in the adult brain, inferring their contribution to neurodegenerative disease pathophysiology and our ability to develop potential therapies to modulate or remove senescent cells from the human brain^25,35^.

One recent attempt to generate a comprehensive list of senescent markers resulted in the SenMayo gene list^6^, which includes 125 genes that are highly correlated with age and the expression level of p16 and p21, cyclin-dependent kinase inhibitors that are upregulated in many senescent cells. The majority of genes in the SenMayo panel are senescence-associated secretory phenotype (SAPS) factors. However, cellular senescence has other aspects, including activation of senescent cell anti-apoptotic pathways (SCAPs)^36^, organelle dysfunction, and morphology changes. To provide a comprehensive and multidimensional perspective for neuronal senescence (neurescent), we employed an eigengene approach based on a) the SenMayo gene list, in addition to our two previously published lists^16^ including b) Canonical Senescence Pathway (CSP) with 22 genes, which reflect cell cycle arrest^1,37,38^, and c) Senescence Initiating Pathway (SIP) panel with 48 genes, which are upregulated in early senescence and activate SCAPs.

Technically, an eigengene is computed as a weighted average expression of all genes in a given list^39,40^ derived using principal component analysis on those particular genes. In this study, we computed an eigengene^40^ for each of the SenMayo, CSP, and SIP gene lists and used all these eigengenes for three main analyses: 1) identifying senescent and non-senescent cells, 2) identifying the most accurate markers for neuronal senescence, referred to as neurescence^34^, and 3) identifying differentially expressed genes in senescent vs non-senescent neurons.

## Results

We used four independent single-nucleus RNA sequencing (snRNA-seq) datasets of the dorsal prefrontal cortex from postmortem human brains were used, which are here referred to as Mathys 2019^41^ (n = 80,000), Zhou 2020^42^ (n = 70,000), Xiong 2023^43^ (n = 400,000), and Mathys 2024^44^ (n = 255,000), respectively (Table 1). Mathys 2019 served as the discovery dataset to identify markers and the other three datasets were used to validate the performance of the identified markers.

Performing the eigengene approach^16^ on the Mathys 2019 discovery dataset, we identified 475 senescent and 16,871 non-senescent neurons (Table 1). Specifically, the identified 475 neurescent cells expressed each of the three CSP, SIP, and SenMayo eigengenes more than the corresponding mean plus three times the standard deviation (mean + 3 s.d.). In contrast, if a neuron expressed each of these eigengenes less than the corresponding mean, then that neuron was considered non-senescent (N = 16,871). The remaining 53,288 borderline neurons were excluded from our analysis. We used the identified neurescent and non-senescent neurons to train decision trees and to perform differential expression (DE) analysis. Of note, only excitatory neurons displayed consistent expression across all three senescence eigengenes, and subsequent differential expression analysis and marker identification were restricted exclusively to excitatory neurons.

To further assess the robustness of our method and the reproducibility of the eigengene-derived senescence markers, we conducted a reciprocal validation analysis. We used Mathys 2024 as the discovery dataset to independently derive the eigengene weights for CSP, SIP, and SenMayo, and then applied these derived weights to Mathys 2019 as a validation dataset. Comparing the weights from the two independent discovery datasets (Mathys 2019 vs. Mathys 2024) (Supplementary Table 1) revealed high concordance: specifically, correlations of 94% for CSP weights, 88% for SIP weights, and 88% for SenMayo weights, respectively (Supplementary Fig. 3). Furthermore, when using the Mathys 2024-derived eigengene weights to identify senescent cells in the Mathys 2019 dataset, we identified 413 senescent neurons, closely matching the original 475 neurons that were identified using Mathys 2019 as discovery. Notably, 370 neurons were identified as senescent in both approaches, representing a highly significant overlap (hypergeometric test p-value < 10^−1714^). Collectively, these results demonstrate the robust consistency and reproducibility of our eigengene-based senescence identification framework, regardless of the initial discovery dataset selection.

### Identification of specific markers for neurons among genes generally associated with senescence

To identify potential markers for neurescence, we fitted relatively small decision trees^45^ to the discovery dataset. To ensure the selected genes were specific, each tree was allowed to use no more than two genes from the pool of gene sets known to be associated with cellular senescence^6,16^, as indicated on the third row of Table 2.

**Figure 1:**
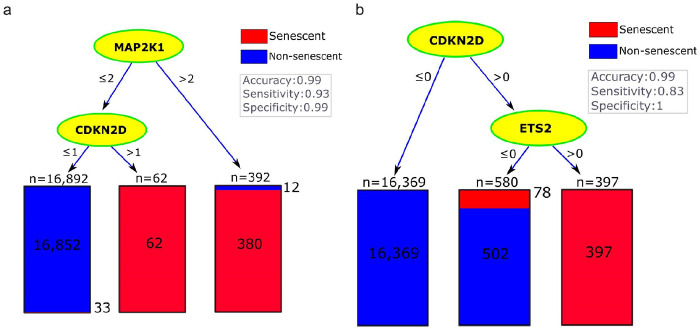
The decision tree analysis based on CSP, SIP, and SenMayo identified senescence markers in neurons. The trees show the classification of neurons into senescent (red) and non-senescent (blue). Each node represents a decision point based on **(a)** gene expression **levels** of **the** original counts, and **(b)** the binary transformed expression. This binary tree reached zero false positive and thus a specificity of 1.v

Using the original counts to quantify gene expression levels, the best marker among the 180 senescence-associated genes was *MAP2K1* leading to a single-gene tree with the highest accuracy of 99%, a sensitivity of 80%, and a specificity of 99% (Table 2, column 4). Adding *CDKN2D* as the second marker increased sensitivity to 93% with a negligible (i.e., < 0.1%) effect on accuracy. The corresponding decision tree, which was based on *MAP2K1* and *CDKN2D*, had the best accuracy among all of our trees that could use two of the 180 senescence-associated genes. This suggested that these two genes could serve as effective markers and complement each other in identifying neurescence. In the corresponding tree, the thresholds for the expression levels of these genes were 2 and 1, respectively (Fig. 1a), suggesting relatively low expression of these putative marker genes. These thresholds are undesirable for histology markers because distinguishing between levels of expression under the microscope is practically challenging. One would prefer markers that are totally absent in negative cells and ideally, have more than one transcript per cell in senescent neurons to ensure the observable signal is above background.

To provide greater confidence in these lowly expressed marker genes, we repeated our analysis using a binary transformation of the discovery data, where any expression level above zero was converted to 1. Using these binary values, no single gene was found to accurately classify the senescent and non-senescent cells. This suggests that there may not be a single marker in our gene lists specifically expressed in neurescence. Interestingly, the best tree with two genes still included *CDKN2D*, but *MAP2K1* was replaced with *ETS2* (Fig. 1b, last row of Table 2). The binary transformation did not change the accuracy but dropped the sensitivity from 93–83%, while increasing the specificity to 100%. After removing *CDKN2D* and *ETS2* from the analysis, *E2F3* and *RB1* were selected in the second best tree (Table 2, column 5), leading to an accuracy of 99%, specificity of 100%, but a relatively low sensitivity of 67%.

### Differentially expressed genes

**Figure 2:**
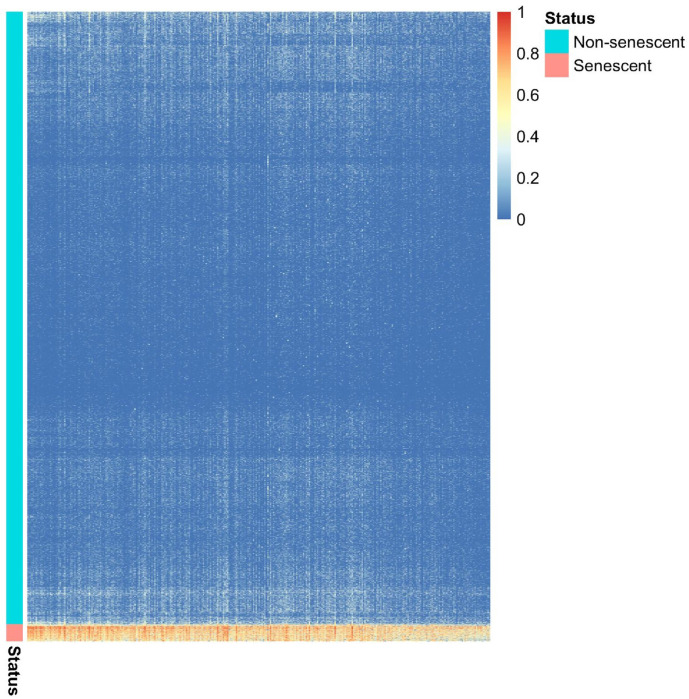
The heatmap of differentially expressed genes identified by comparing senescent versus non-senescent neurons. The heatmap shows the expression profile of 324 genes (columns) in the 17,346 cells (rows) in the discovery dataset. These genes were consistently identified as differentially expressed by both MAST and SigEMD methods.

To investigate differentially expressed genes in neurescence compared to non-senescent, we performed a DE analysis on the Mathys 2019 discovery dataset. Taking an agnostic approach, we included all 10,768 genes that had non-negligible expression in neurons of the discovery dataset (Methods). We employed two state of the art methods for DE analysis of scRNA-Seq data. The first method, MAST^46^, resulted in 375 differentially expressed genes, whereas using the second method, SigEMD^47^, we identified 576 differentially expressed genes (Supplementary Table 2). The two analyses shared 324 differentially expressed genes (Fig. 2, Supplementary Table 2), which represented a significant overlap (p-value < 10^−205^, hypergeometric test). These 324 genes were overexpressed in the 475 neurescent cells compared to other neurons (Fig. 2). In this study, we primarily focused on these 324 genes because they were identified by both DE analysis methods.

### Pathway analysis

**Figure 3:**
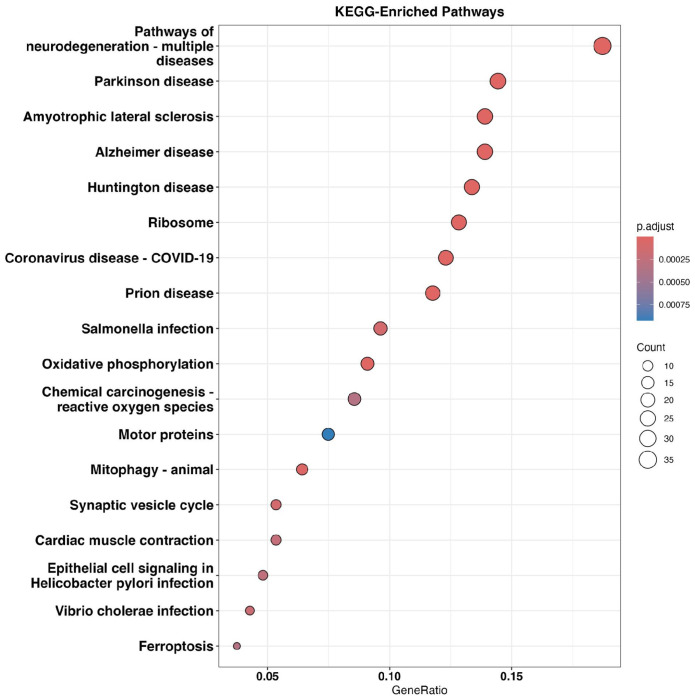
Pathways overrepresented by genes that are differentially expressed in senescent neurons. The bubble chart displays the significantly enriched pathways identified from the pathway analysis of 324 differentially expressed genes. Each bubble represents a pathway, with the size indicating the number of DE genes in that pathway and the color denoting the adjusted p-value for overlap significance.

To understand the functional significance of the 324 differentially expressed genes, we performed a pathway analysis using Kyoto Encyclopedia of Genes and Genomes (KEGG)^48^ and identified 18 significantly enriched pathways (p-value < 0.05, Fig. 3). Interestingly, the top pathways were related to neurodegeneration including Parkinson disease^49,50^, Amyotrophic lateral sclerosis^51^, Alzheimer’s disease^52,53^, and Huntington’s disease^54^, suggesting common underlying biological mechanisms that might be associated with neuronal senescence.

**Figure 4:**
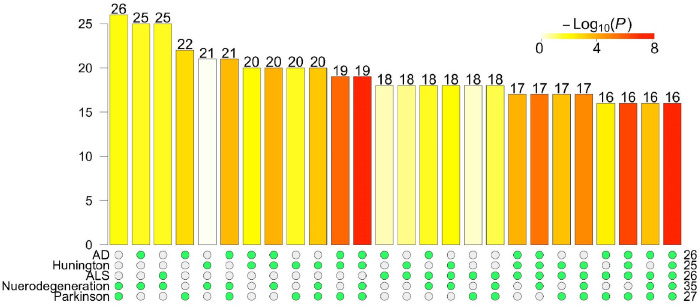
Number of DE genes in the top enriched pathways. The number of DE genes in each pathway is listed on the right. The UpSet^62^ plot shows the sizes of the overlaps among DE genes and the top five significantly enriched pathways named on rows. In particular, the last column shows 16 DE genes are members of all these five pathways, which is a significant overlap (p-value < 10^−8^).

The top 5 enriched pathways shared 16 DE genes, which is a significant overlap (p-value < 10^−8^, hypergeometric test, Fig. 4). These genes included *COX6C*, *COX7A2L*, *COX7C*, *CYCS*, *KIF5A*, *NDUFA4*, *NDUFS1*, *PSMA7*, *TUBA4A*, *TUBB2A*, *TUBB4A*, *TUBB4B*, *UQCRB*, *UQCRC2*, *UQCRH*, and *UQCRHL*. These 16 genes are mainly involved in mitochondrial function (COX, NDUF families)^55,56^ and cytoskeletal structure (TUBB family)^57,58^. Mitochondrial dysfunction leads to impaired energy metabolism and increased production of reactive oxygen species^59^. It is a hallmark of both cellular senescence^60^ and AD^61^. The deregulation of these genes supports prior work demonstrating mitochondrial dysfunction in neurescence, and their role in the neurodegeneration^18^.

### Identification of specific markers for senescent neurons among differentially expressed genes

**Figure 5:**
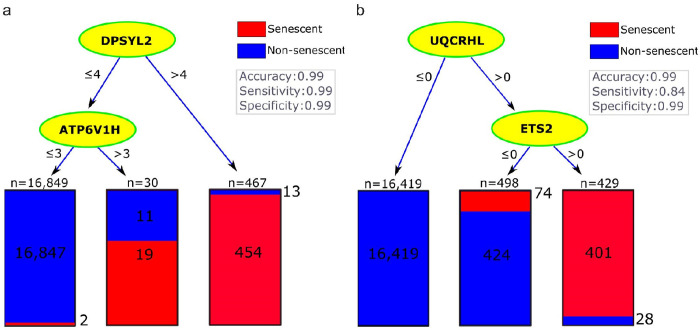
Decision tree analysis based on CSP, SIP, SenMayo, and DE genes identified novel senescence markers in neurons. The trees show the classification of neurons into senescent (red) and non-senescent (blue) using a pair of selected markers from a pool of differentially expressed genes merged with CSP, SIP, and SenMayo gene lists. Each node represents a decision point based on **(a)** gene expression thresholds of original counts, and **(b)** binary transformed expression.

We hypothesized that there might be some specific markers for neurescence beyond the genes generally known to be associated with senescence (e.g., CSP, SIP, and SenMayo). To test this hypothesis, we extended our decision tree analysis to include the 324 differentially expressed genes identified based on the discovery dataset. Using the original count data, the top gene was *DPYSL2* resulting in a relatively accurate classification (i.e., accuracy: 99%, sensitivity: 95%, and specificity: 99%) and when the gene *ATP6V1H* was added to make a two-gene tree, sensitivity increased to 99% (Table 2, column 6). However, for these trees to be useful, meaning that they identify molecules that can serve as biomarkers in histological assays, one must be able to determine whether these genes were expressed at relatively high levels in a neuron (i.e., above 4 and 3, respectively) (Fig. 5a). To address this practical issue, we used binary expression levels leading to selection of *UQCRHL* together with *ETS2* (Fig. 5b). The recurrent selection of *ETS2* (Table 2, columns 4 and 6) highlighted its importance even when additional differentially expressed genes were added to the analysis. Since the inclusion of *UQCRHL* led to very low sensitivity in the validation cohorts (Table 3), we considered removing this gene. Excluding *UQCRHL* and *ETS2* led to selection of *CDKN2D* and *RB1* again and increasing the specificity of senescence classification to 100% in the discovery dataset. Overall, adding differentially expressed genes did not seem to be helpful as the only reasonable tree in columns 6 and 7 was based on *CDKN2D* and *RB1*, which were already in our CSP gene lists. This observation falsified the hypothesis that additional differentially expressed genes could identify specific markers for senescent neurons.

### Identification of alternative markers for senescent neurons

The relatively low expression of the selected markers, combined with historical challenges in developing antibodies specific to senescence markers, necessitates that *in silico* studies identify multiple candidate molecules for subsequent *in situ* validation. We performed a systematic elimination analysis. We pooled all genes from CSP, SIP, and SenMayo lists with DE genes and fitted decision trees on the binary expression values in the discovery dataset, Mathys 2019 (Table 3). Our systematic approach involved iteratively removing the top-performing markers to evaluate their impact on model performance and to identify the next best set of markers.

Including *UQCRHL* in decision trees led to poor sensitivity in the validation datasets. Thus, we excluded *UQCRHL* and found *CDKN2D* and *ETS2* to be the second best pair. If reagents to detect *ETS2* are unavailable or lack specificity, then *CDKN2D* and RB1 would be the next best pair of markers for neurescence. Our results showed that removing *CDKN2D* generally led to decreased accuracy, sensitivity, and specificity in validation datasets. This drop in model performance highlights the critical role *CDKN2D* plays in the classification. However, it is important to note that the currently available antibodies for p19, the protein product of *CDKN2D*, may lack specificity, which could complicate its use in histological applications.

## Discussion

Identifying senescent neurons in the human brain is challenging due to the inherent heterogeneity of senescence, neuronal subtypes and the current lack of reliable markers specific to neurescence in the human brain^63^. To tackle this challenge, we systematically and unbiasedly analyzed scRNA-seq data from four datasets of the dorsal prefrontal cortex from postmortem human brains. Our eigengene-based method allowed us to focus on three key gene lists: SenMayo, CSP, and SIP, each reflecting distinct aspects of cellular senescence.

While the recently published SenMayo list is a valuable resource, it primarily captures the inflammatory aspects of senescence. The majority of genes in this list are associated with Senescence-associated secretory phenotype and other important characteristics of neural senescence may not be represented by SenMayo. Inflammation is a critical component, particularly in the brain, where it has been linked to neurodegeneration^64,65^. The accumulation of senescent cells can lead to a persistent pro-inflammatory state, driven by mitochondrial dysfunction ^66,67^, and concomitant elevated levels of actors like IL-6, IL-1β, and TNF-α, which are characteristic of SASP^68–70^. This chronic inflammation can impair tissue homeostasis and contribute to neuronal dysfunction^71^. However, inflammation alone does not fully capture the multifaceted nature of cellular senescence, where other mechanisms such as cell cycle arrest, DNA damage, mitochondrial dysfunction, and metabolic alterations also play critical roles^63,72,73^. Moreover, inflammation can occur independent of senescence. This inherent limitation of the SenMayo list underscores the need for a more comprehensive approach. Accordingly, in this study, we included CSP and SIP gene panels to capture other important senescence features such as cell cycle arrest and early stress responses. Our approach leveraged eigengenes derived from these three panels to classify neurons into senescent and non-senescent groups. Our integrative approach using multiple senescence-associated gene panels aims to improve specificity by capturing their combined activity rather than relying on any single pathway.

Our findings demonstrated that a single marker gene is insufficient for accurately classifying neurescence. Decision tree analysis, incorporating multiple senescence panels and both continuous and binary expression data, revealed that combining markers significantly improves classification performance. For example, using *ETS2* with *CDKN2D* enhanced model sensitivity and specificity significantly (Table 3, Supplementary Fig. 2), highlighting the complex signature of neurescence that cannot be captured by a single gene. While finding reliable markers for neurescence remains a difficult task, our paired-marker strategy, particularly using genes like *CDKN2D* and *ETS2*, can offer a promising direction for future studies on neurescence.

As expected, pathways associated with neurodegeneration are upregulated in neurescence. Interestingly, we identified 16 DE genes that are overexpressed in all of these upregulated pathways. These 16 genes are related to mitochondrial function and cytoskeletal structure. While the association between mitochondrial function and neurodegeneration has been known^59,74–80^, our contribution is to show that these pathways are enriched specifically in neurescence. Follow-up studies to elucidate the biological mechanisms linking these genes and mitochondrial function and neurodegeneration could facilitate the discovery of novel therapeutic targets for AD. Our findings can help such studies focus on mitigating the impact of cellular senescence on neurodegeneration.

This study provides promising markers for identifying neurescence, albeit some limitations need attention. Using the original counts to fit decision trees led to non-zero thresholds, which are undesirable for histology markers because distinguishing between levels of expression under the microscope is practically challenging. One would prefer markers that are totally absent in negative cells and ideally, have more than one transcript per cell in senescent neurons to ensure the observable signal is above background. Therefore, we used the binary values.

Positive signals using antibodies against cyclin-dependent kinase inhibitors associated with senescence have historically been unreliable ^81^. *CDKN2D*, cyclin-dependent kinase 4 inhibitor, encodes p19^INK4D^, which we found to be elevated in NFT-bearing neurons in a prior study ^16^ However, that study revealed that not all neurescent cells expressed p19^INK4D^, and not all p19^INK4D^-positive cells were neurescent. We hypothesize that co-staining with antibodies against p19^INK4D^ and *ETS2* may increase specificity of neurescence identification, but this remains to be experimentally determined.

**Figure 6:**
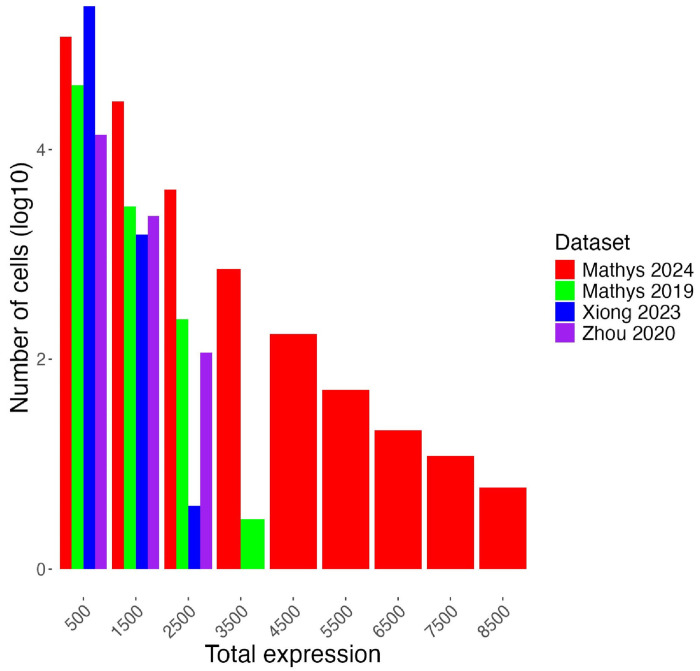
Distribution of total expression of 499 senescence marker genes. The plot illustrates the inherent variability in gene expression distributions among datasets derived from postmortem human brain samples (Table 1). The x-axis represents the sum of expression values of all marker genes in each cell. The x-axis is grouped into bins of size 1000. While Mathys 2019 (green) and Mathys 2024 (red) exhibit a more even distribution of gene expression, Zhou 2020 (purple) and Xiong 2023 (blue) display skewed distributions towards sparser expression.

Another limitation working with snRNA-seq data lies in the inherent variability among the datasets on hand, despite all being derived from the same region of postmortem human brains. For instance, the distribution of total expression of senescence markers per cell varies considerably across the four datasets (Fig. 6). In particular, Mathys 2019 and Mathys 2024 display a more even distribution of gene expression across different levels, while Zhou 2020 and Xiong 2023 exhibit a more skewed distribution towards sparser expression. These differences highlight the challenge of comparing datasets generated by different research groups and under different experimental conditions. The observed variability may stem from technical error, different sample handling procedures, instruments, or biological factors such as the heterogeneity of sampled brain regions and variable progression of neurodegenerative diseases in donors. Also, while our study focused on neuronal senescence, it is important to note that other brain cell types, such as astrocytes, microglia, and oligodendrocyte-lineage cells, have also been reported to exhibit senescent-like phenotypes ^82–84^. Given the likely variation in senescence marker profiles across these cell types, future studies applying similar approaches to characterize non-neuronal senescence will be essential for the development of targeted senolytics in the brain. Addressing these challenges could pave the way for expanding this study to include more diverse datasets, including those from earlier disease stages and other brain regions. Such an approach would help validate the robustness of the markers identified here.

## Methods

### The snRNA-Seq datasets

We used four snRNA-Seq datasets generated by Mathys et al.^41^, Zhou et al.^42^, Xiong et al.^43^, and Mathys et al.^44^, which are accessible through the Accelerating Medicines Partnership–AD (AMP–AD)^85^ with synapse IDs syn18485175, syn2112584, syn52293424, and syn52293442, respectively. The former dataset, referred to as Mathys 2019, was used in all analyses in this study as the discovery (i.e., train) dataset. The latters, referred to as Zhou 2020, Xiong 2023, and Mathys 2024 respectively, were used to validate the robustness and sensitivity of the proposed markers and our decision tree models. All samples were originally generated by longitudinal clinical-pathologic cohort studies of aging and Alzheimer’s disease (AD) from the Religious Order Study (ROS) and the Rush Memory and Aging Project (MAP)^86^. We employed the Synapser (https://r-docs.synapse.org/articles/synapser.html) R package^87^ (Version 0.6.61) and a custom R script (Version 4.4.1) to download the four snRNA-seq datasets^41–44^. We downloaded clinical data from the corresponding publication pages. The snRNA-seq datasets included approximately 80,000, 70,000, 400,000, and 1.3 million single nuclei samples, with median postmortem interval (PMI) of 7, 6, 6, and 6 hours, respectively. We included only excitatory and inhibitory neurons in our analysis (Table 1). The first three datasets used samples from the dorsal prefrontal cortex of 48, 32, and 92 postmortem human brains, respectively. The Mathys 2024 dataset expanded the scope to include samples from entorhinal cortex (EC), hippocampus (HC), anterior thalamus (TH), angular gyrus (AG), midtemporal cortex (MT), and prefrontal cortex (PFC) regions of the same brain across 48 postmortem human samples. In this study, we used only the PFC samples, which included approximately 255,000 single nuclei transcriptomes.

### Eigengene analysis

An eigengene for a given set is the first principal component (PCA)^88^, which is the weighted average of expression of all genes in the set. For each of the three SenMayo, CSP, and SIP gene lists, we used the compute.pigengene() function from the Pigengene package (Version 1.30.0)^40^ to compute an eigengene. We addressed the challenge of cell type imbalance by implementing weighted PCA using the weight.pca() function from the Pigengene package to ensure that each cell type contributed equitably to the analysis. Specifically, each cell was weighted by dividing the total number of cells by the frequency of the corresponding cell type. This approach assigns higher weights to rarer cell types and lower weights to more abundant ones, thus balancing their influence in the PCA. We used the project.eigen() function from the Pigengene package to infer eigengene values in the validation datasets. This function computes each eigengene in the validation dataset using the same weights learned from the discovery dataset. After the weighted average is computed, the inferred eigengene was normalized to have the same Euclidean norm as the original eigengene.

### Cell labeling

Three eigengenes were computed based on three independent gene sets associated with senescence: (1) Canonical Senescence Pathway (CSP)^16^, (2) Senescence Initiating Pathway (SIP)^16^, and (3) SenMayo^6^, which consisted of 22, 48, and 125 genes, respectively. The actual number of genes contributed to our eigengene analysis was slightly lower because the discovery dataset included only 22, 44, and 116 of these genes, respectively. We calculated the mean and standard deviation of each eigengene based on the empirical distribution in the discovery dataset. Any cell expressing an eigengene more than mean plus three standard deviations (i.e, mean + 3 s.d.) was considered “overexpressing” the eigengene, and was labeled as CSP^+^, SIP^+^, or SenMayo^+^, depending on their respective gene set. In contrast, any cell expressing the eigengene below the mean was labeled as CSP^−^, SIP^−^, and SenMayo^−^, respectively. Cells overexpressing all the three eigengenes were labeled as “senescent”, indicating a consensus across the three gene sets. In contrast, cells were labeled “non-senescent” when they expressed all the three eigengenes less than the corresponding means (Table 1). Other cells that did not meet either of these two criteria were considered borderline and excluded from our analysis. We used the phyper() function from the stats R package (Version. 4.4.1)^87^ to perform a hypergeometric test with the null hypothesis that the number of senescent cells observed in a cell type is more than what would be expected at random.

### Differential expression analysis

Our DE analysis was based on the senescent neurons that were overexpressing all three eigengenes (above mean + 3 s.d.) compared to the non-senescent neurons (below mean) (Table 1). We also filtered out 7,158 genes that did not have the minimum expression of 1 in at least 200 neurons. We normalized the data by multiplying all nuclei counts by the total library size of 1 million and transformed it to logarithmic space in base 2 (log_2_). Given the significant imbalance between the groups, the smaller senescent class was upsampled by repeating each neuron 36 times so that the the number of non-senescent and senescent neurons appeared roughly equal for the DE analysis. We performed the differential expression analysis for senescent and non-senescent neurons, using two popular methods developed for scRna-seq, and the overlapped genes were chosen as the final differentially expressed genes. The first method was carried out using the MAST package (Version 1.30.0)^46,89^, which implemented a hurdle model^89^ for analyzing scRNA-seq data that consists of a two-part generalized linear model. Considering the bimodality characteristic in single-cell expression data, MAST jointly models the positive mean expression (continuous) and the rates of expression (discrete) values. In this method, genes with a false discovery rate (i.e., adjusted p-value) less than 0.01 and an absolute value of log_2_ fold change above 6, were declared as differentially expressed genes. The second method, SigEMD (Version 0.21.1)^47,90^, which is a custom R script that uses the nonparametric Earth Mover’s Distance (EMD)^91^. EMD is a special case of Wasserstein metric^92^, and measures the distance between gene expression distributions. Accordingly, a predefined adjusted p-values under 0.01 and an EMD score more than 30 were set to identify differentially expressed genes in senescent vs non-senescent neurons. Heatmap of differentially expressed genes was generated using the pheatmap.type() function from the Pigengene R package^40,93^.

### Decision trees

Our primary objective in fitting decision trees to the discovery dataset was to identify the most predictive markers. We used the C50 package (Version 0.1.8)^94^ in R to construct multiple decision trees based on different criteria. These trees differ in two ways: a) the eigengenes that served as the basis for neuron labeling and b) the selection of genes used as features in each tree (Table 2). To prevent overfitting, we set the parameter minCases of the function C5.0() to relatively large values to control the number of genes that contribute to a decision tree. This parameter specifies the minimum number of samples required in at least two splits of the tree, thus a larger minCases value results in a smaller decision tree. In this way, none of our trees was allowed to select more than two genes from the pool of genes available to it.

We assessed whether the marker genes selected by the decision tree trained on the discovery dataset could accurately classify neurons in the validation datasets. The predict() function was applied on validation datasets to derive performance metrics of the decision trees fitted to the discovery dataset. This function predicted the senescence status of each neuron based on the trees fitted to the discovery dataset and the expression levels in the validation datasets. Particularly, the same cutoff values on gene expression values that were learned from the discovery dataset were used for the validation dataset. The predictions by decision trees, which used a few single genes, were then compared to the eigengene-based senescence labels. This comparison was done in each of the validation sets to compute accuracy, sensitivity, and specificity^95^ of the trees (Table 3). Specfically, the neurons that were identified senescent by both a decision tree and eigengene approaches were considered true positives. In contrast, true negatives were the neurons that were not senescent based on both approaches. In each dataset, a) accuracy was defined as the number of true positives over the total number of neurons in the dataset, b) sensitivity was defined as the number of true positives over the number of neurescents based on our eigengene approach, and c) specificity was the ratio of true negatives over the total number of neurons that were not senescent based on our eigengene approach.

The trees varied in the labels used to train them and also in the pool of genes they could select from. Initially, for each of the CSP, SIP, and SenMayo gene lists, we calculated their corresponding eigengenes, along with the positive and negative labels. Note that the resulting labelings could differ across gene lists. Using these three eigengenes and their corresponding gene lists, we trained three independent decision trees (Table 2, columns 1–3). Each tree specifically represented the unique characteristics derived from one of the gene lists.

We trained a fourth decision tree using the senescent and non-senescent neuron labels unanimously defined based on all of our three eigengenes (Methods, Cell labeling) (Table 1). The fourth tree was allowed to use any of the 180 unique genes from the pooled set of all the genes in the CSP, SIP, and SenMayo lists (Table 2, columns 4 and 5). To train the fifth tree, we used the same senescent and non-senescent labels utilized in the fourth tree, but expanded the gene pool to include the 324 differentially expressed genes in addition to the previously combined CSP, SIP, and SenMayo gene lists. This led to including a total of 499 unique genes for training the fifth tree (Table 2, column 6 and 7).

Furthermore, our methodology involved training the decision trees twice: first using the original counts directly from the expression matrix that quantified expression levels, and secondly by converting the expression levels into binary values, where any expression level above zero was considered 1.

### Pathway enrichment analysis

We performed a pathway enrichment analysis based on the Kyoto Encyclopedia of Genes and Genomes^48^ (KEGG) database using the get.enriched.pw() function from the Pigengene R package (Version 1.30.0)^40,96,97^. In this way, we identified the pathways that were overrepresented (adjusted p-values of the hypergeometric test < 0.05) by the genes that were differentially expressed between senescent and non-senescent neurons. Furthermore, we used the supertest() function from SuperExactTest R package (Version 1.1.0)^62^ to assess the statistical significance of overlapping genes among multiple pathways.

## Data Availability

The snRNA-Seq data analyzed in this study are available from https://www.synapse.org/ with synapse IDs: syn18485175, syn2112584, syn52293424, and syn52293442 for Mathys 2019, Zhou 2020, Xiong 2023, and Mathys 2024, respectively. Accessing these data requires submitting a Data Use Certificate through the AMP–AD website. Clinical data were available in the corresponding publication pages.
